# Productive HBV infection of well-differentiated, hNTCP-expressing human hepatoma-derived (Huh7) cells

**DOI:** 10.1007/s12250-017-3983-x

**Published:** 2017-09-29

**Authors:** Ming Zhou, Kaitao Zhao, Yongxuan Yao, Yifei Yuan, Rongjuan Pei, Yun Wang, Jizheng Chen, Xue Hu, Yuan Zhou, Xinwen Chen, Chunchen Wu

**Affiliations:** 1 State Key Laboratory of Virology, Wuhan Institute of Virology, Chinese Academy of Sciences, Wuhan, 430071 China; 2 Shenzhen Xenotransplantation Research and Development Center, State and Local Joint Cancer Genome Clinical Application of Key Technology Laboratory, Shenzhen Second People’s Hospital, First Affiliated Hospital of Shenzhen University, Shenzhen, 518035 China; 3 Institute of Immunology, Zhongshan School of Medicine, Guangdong Provincial Key Laboratory of Organ Donation and Transplant Immunology, Sun Yat-sen University, Guangzhou, 510080 China

**Keywords:** Hepatitis B virus (HBV), Na+/taurocholate cotransporting polypeptide (NTCP), Huh7, dimethyl sulfoxide (DMSO), polyethylene glycol (PEG), susceptibility

## Abstract

Feasible and effective cell models for hepatitis B virus (HBV) infection are required for investigating the complete lifecycle of this virus, including the early steps of viral entry. Resistance to dimethyl sulfoxide/polyethylene glycol (DMSO/PEG), hNTCP expression, and a differentiated state are the limiting factors for successful HBV infection models. In the present study, we used a hepatoma cell line (Huh7D^hNTCP^) to overcome these limiting factors so that it exhibits excellent susceptibility to HBV infection. To achieve this goal, different hepatoma cell lines were tested with 2.5% DMSO / 4% PEG8000, and one resistant cell line (Huh7D) was used to construct a stable hNTCP-expressing cell line (Huh7D^hNTCP^) using a recombinant lentivirus system. Then, the morphological characteristics and differentiation molecular markers of Huh7D^hNTCP^ cells with or without DMSO treatment were characterized. Finally, the susceptibility of Huh7D^hNTCP^ cells to HBV infection was assessed. Our results showed that Huh7D cells were resistant to 2.5% DMSO / 4% PEG8000, whereas the others were not. Huh7D^hNTCP^ cells were established to express a high level of hNTCP compared to liver extracts, and Huh7D^hNTCP^ cells rapidly transformed into a non-dividing, well-differentiated polarized phenotype under DMSO treatment. Huh7D^hNTCP^ cells fully supported the entire lifecycle of HBV infection. This cell culture system will be useful for the analysis of host-virus interactions, which should facilitate the discovery of antiviral drugs and vaccines.

**Electronic Supplementary Material:**

Supplementary material is available for this article at 10.1007/s12250-017-3983-x and is accessible for authorized users.

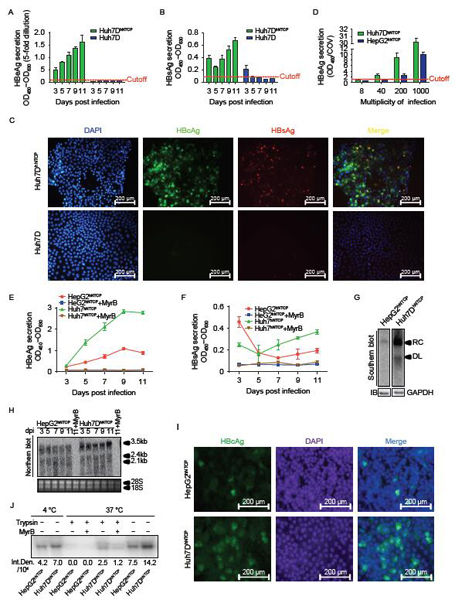

## INTRODUCTION

Hepatitis B virus (HBV) infection causes a wide spectrum of clinical manifestations ranging from an asymptomatic carrier state to acute or chronic hepatitis, with progression to liver cirrhosis and hepatocellular carcinoma. Currently available interferon and nucleos(t)ide analogues (NA) cannot effectively eradicate the virus. A better understanding of the HBV life cycle is necessary to design more effective therapeutic strategies.

Feasible and effective cell models for HBV infection are required for investigating the complete lifecycle of this virus, including the early steps of the viral cycle. Currently, primary human hepatocytes (PHHs) (Lempp et al., 2017; Ni and Urban, 2017; Shimura et al., 2017), HepaRG cells (Gripon et al., 2002; Schulze et al., 2012), and primary *tupia* hepatocytes (PTHs) (Kock et al., 2001; Ren and Nassal, 2001) are the main cell models used to study the early steps of HBV infection. PHHs, the natural host of HBV, are regarded as the ideal cellular model for HBV infection. However, poor accessibility, lot-to-lot variation, and rapid loss of susceptibility limit the applications of PHHs. HepaRG cells, a human liver progenitor cell line, retain the differential ability to polarize into hepatocyte-like cells and become susceptible to HBV infection following induction with dimethyl sulfoxide (DMSO) and hydrocortisone (Gripon et al., 2002). The drawbacks of HepaRG cells, such as low efficiency of infection, a time-consuming process for the induction of PHH-like polarization, and patent protection, prevent their wide utilization. PTHs, an alternative for PHHs, can be accessed more easily, and more homogeneous populations can be obtained. However, the addition of DMSO and polyethylene glycol 8000 (PEG8000) during the infection of PTHs does not significantly promote the infectivity of HBV (Weizsäcker and Roggendorf, 2005). In addition, human serum competes with HBV particles for binding to the cell membrane (Kock et al., 2001), leading to a reduced experimental confidence level. Novel HBV infection models are thus urgently needed.

In 2012, a functional receptor for HBV/HDV infection, Na+/taurocholate cotransporting polypeptide (NTCP) (Yan et al., 2012), was identified. A 3.6-fold induction of hNTCP mRNA was observed in HepaRG cells differentiated by DMSO exposure, whereas knockdown of hNTCP was shown to completely block HBV and HDV infection in HepaRG cells, suggesting the essential role of hNTCP in HBV/HDV entry (Ni et al., 2014). A HepG2 cell line stably expressing hNTCP (HepG2$^{hNTCP}$) was reported to support HBV infection (Yan et al., 2012; Ni et al., 2014).

As HBV naturally infects and replicates in highly differentiated and non-dividing human hepatocytes, cells in a non-dividing, well-differentiated state may be superior to poorly differentiated and dividing hepatoma cells for establishing HBV infection (Sainz and Chisari, 2006; Tian et al., 2016). DMSO enhances cell differentiation and HBV transcription and replication, and it is normally added to the culture media of HBV-infected PHHs and HepaRG cells (Gripon et al., 1988; Gripon et al., 2002; Schulze-Bergkamen et al., 2003). However, different cells exhibit quite different tolerances to DMSO. Therefore, DMSO and a differentiated state are regarded as limiting factors for establishing feasible and effective HBV infection models. In addition, resistance to 4% PEG8000 and hNTCP expression level are also limiting factors for effective HBV infection (Gripon et al., 1993; Yan et al., 2012).

Huh7 is a hepatoma cell line originally obtained from a 57-year-old Japanese male in 1982 who was suffering from liver tumor (Nakabayashi et al., 1982). After induction by 1% DMSO, Huh7 can transform into a non-dividing, well-differentiated phenotype (Sainz and Chisari, 2006), which may be a feasible platform to establish productive HBV infection. In the present study, we established an hNTCP-expressing Huh7 cell line (Huh7D$^{hNTCP}$) that was tolerant to 2.5% DMSO/4% PEG8000, stably expressed a high level of hNTCP, and rapidly transformed into a non-dividing, well differentiated phenotype following DMSO induction. Under these conditions, Huh7D$^{hNTCP}$ cells possessed an optimized susceptibility to HBV infection.

## MATERIALS AND METHODS

### Human materials and ethical statement

Embryonic liver tissues were kindly provided by Zhongnan Hospital of Wuhan University with informed written consent from all participating family members. Ethical approval for the study was granted by the institutional bioethics committee of Wuhan Institute of Virology, Chinese Academy of Sciences (approval number WIVH 24201101).

### Vector construction and lentivirus production

The lentiviral vectors (pWPI) and packaging plasmids (pCMV-dR8.91 and pMD.2G) were obtained from Addgene (http://www.addgene.org) and have been described elsewhere (Wiznerowicz and Trono, 2003). To construct pWPI-Puro, the puromycin-resistance gene was inserted to replace GFP. hNTCP (with the same coding sequence as previously reported) (Yan et al., 2012) was inserted into pWPI-Puro. Recombinant lentiviruses were produced as previously described (Wang et al., 2013). After 48 h, the culture medium was collected, and centrifuged to remove debris at 1000 × *g* for 10 min, followed by filtration through 0.45-μm cellulose acetate filters (Millipore, Billerica, Massachusetts, USA). Then, the filtered medium was centrifuged using Ultracel-100K centrifugal filters (Millipore). Finally, the concentrated lentivirus was stored at –80 °C ready for experiments.

### Cell culture and cell establishment

Huh7D$^{hNTCP}$ cells were established by adding recombinant lentivirus to the culture medium in the presence of 6 μg/mL polybrene (Sigma, Shanghai, China). The medium was refreshed 12 h later, and 5~10 μg/mL puromycin (InvivoGen, San Diego, CA, USA) was added to the cells 48 h later to screen for positive cells.

Cell lines (293T, HepG2, Huh7, Huh7D$^{hNTCP}$, and HepG2$^{hNTCP}$) were maintained in Dulbecco's modified Eagle's medium (DMEM, Gibco, Grand Island, USA) supplemented with 10% fetal bovine serum (Gibco),

100 U/mL penicillin, and 100 μg/mL streptomycin. Huh7D$^{hNTCP}$ cells were induced by 2.5% DMSO (Inducing medium: DMEM supplemented with 5% FBS, 100 U/mL penicillin, 100 μg/mL streptomycin) for 24 h before HBV infection, whereas HepG2$^{hNTCP}$ cells were maintained in PMM (2.5% DMSO) for 24 h before HBV infection, as described elsewhere (Yan et al., 2012).

## HBV virus preparation and HBV/HCV infection

HBV infectious particles were prepared from HepAD38 cells as previously described (Schulze et al., 2010). Concentrated HBV stocks diluted in PMM and supplemented with 4% (w/v) PEG8000 were added directly to susceptible cells. After a 24-h incubation, the cells were washed extensively three times with PBS, and the media were refreshed. Huh7D$^{hNTCP}$ cells were maintained in inducing medium, and HepG2$^{hNTCP}$ cells were maintained in PMM (2.5% DMSO). For the HBV binding and uptake assay, approximately $2 \times 10^6$ Huh7D$^{hNTCP}$ or HepG2$^{hNTCP}$ cells in each well of a 6-well plate were incubated with HBV in the presence of 4% PEG8000 at 4 °C for 24 h (HBV binding only), at 37 °C for 24 h (binding and uptake), or at 37 °C for 24 h but digested with trypsin for 10 min followed by washing twice with 1×PBS to remove bound HBV particles (uptake only). HBV DNA was extracted using a method described by Hirt (Hirt, 1967). Myrcludex B, the analogue of myr-preS1$^{2\text{-}47}$, was synthesized by GL Biochem Company (Shanghai, China) and used to specifically block HBV infection.

HCV J399EM (genotype 2a) was prepared and infected as previously reported (Han et al., 2009; Cao H. et al., 2014a). The HCV virion was added to susceptible cell lines at a multiplicity of infection (MOI) of 1, and chimeric HCV-GFP was recorded with a fluorescence microscope.

## Enzyme-linked immunosorbent assay

The culture media were collected at the indicated time points. The level of HBsAg or HBeAg was detected by enzyme-linked immunosorbent assay (ELISA) according to the manufacturer's instructions (KHB, Shanghai, China). Each sample was measured in triplicate utilizing an Epoch Microplate Spectrophotometer (Bio-Tek, Winooski, USA), and the values are presented as the mean of $OD_{450} - OD_{630}$ ± standard deviation. The cut-off value was calculated according to manufacturer's instructions.

## RNA extraction and reverse transcription for real-time PCR

Total RNA was extracted from the cells, using TRIzol Reagent (Invitrogen, Carlsbad, USA) according to the manufacturer's protocol. Real-time PCR was performed on an ABI QuantSudio 6 Flex device, using a QuantiTech SYBR Green RT-PCR Kit (Qiagen, Hilden, Germany). β-Actin was served as the internal control for sample normalization. The relative mRNAs level was calculated using the $\triangle\triangle C_T$ method. All samples were measured in triplicate, and all experiments were repeated independently three times. The primers used for real-time PCR are listed in Table 1.

## Western blot

Western blot was performed as previously described (Zhou et al., 2014b). The primary antibodies used were rabbit anti-human hNTCP polyclonal antibody (Sigma: HPA042727) and mouse anti-human GAPDH monoclonal antibody (Proteintech™, Chicago, USA).

## Immunofluorescence assay

An immunofluorescence assay was performed as described previously (Zhou et al., 2014b). The primary antibodies were rabbit anti-HBcAg polyclonal antibody (Dako, Glostrup, Denmark), mouse anti-HBsAg monoclonal antibody (Cao L. et al., 2014), mouse anti-CD81 antibody (Santa Cruz, California, USA), and rabbit anti-hNTCP polyclonal antibody (Sigma: HPA042727). The secondary antibodies were Alexa Fluor-488 or Alexa Fluor-568 conjugated (Life Technologies, Waltham, Massachusetts, USA). The nucleus was stained with DAPI (Roche, Basel, Switzerland). The stained cells were examined under a fluorescence microscope (Leica, Wetzlar, Germany) or confocal microscope (PerkinElmer, Massachusetts, USA).

## Southern blot and Northern blot

HBV total DNA was extracted by a method described by Hirt (Hirt, 1967), and core-associated HBV DNA was extracted by a method described elsewhere (Wu et al., 2010). Southern blot was performed as described previously

Table 1. Primers used for real-time PCR

| Primers | Sequence (5′–3′) |
|---|---|
| hNTCP-F | CTCAAATCCAAACGGCCACAATAC |
| hNTCP-R | CACACTGCACAAGAGAATGATGATC |
| HNF4α-F | GGCCAAGTACATCCCAGCTT |
| HNF4α-R | TCATTGCCTAGGAGCAGCAC |
| Albumin-F | ACTATCTATCCGTGGTCCTGA |
| Albumin-R | TCTTGATTTGTCTCTCCTTCT |
| CYP 3A4-F | TCCATTCCTCATCCCAATTCTTGA |
| CYP 3A4-R | TCCACTCGGTGCTTTTGTGT |
| AFP-F | CCAACAGGAGGCCATGCTT |
| AFP-R | GAATGCAGGAGGGACATATGTTT |
| β-actin-F | ATCGTGCGTGACATTAAGGAG |
| β-actin-R | GGAAGGAAGGCTGGAAGAGT |

(Zhou et al., 2014a). Northern blot was performed as described elsewhere (Pei et al., 2014) using a Northern Max Kit (Life Technologies) according to the manufacturer's instructions. Approximately 30 μg denaturized RNA per sample was loaded onto a 1.2% RNase-free agarose gel.

## RESULTS

### Resistance to DMSO and PEG8000

To obtain a Huh7 cell line tolerant to a high concentration of DMSO, we screened four Huh7 cell lines originated from different preservers. On day 4 post-2.5% DMSO treatment, all Huh7 cell lines proliferated to a confluent monolayer, but only the No.1 Huh7 cell line began to show a morphologically polarized character (Supplementary Figure S1). On day 8 post-DMSO treatment, only the No.1 Huh7 line grew well and further developed polarization, whereas the others nearly died out. The changes in cell viability were further confirmed using a CCK8 assay (Figure 1A). To discriminate it from the other three Huh7 cell lines, the No.1 Huh7 line was named Huh7D.

Normally, 4% PEG8000 is added to the incubation medium during infection to enhance HBV attachment (Gripon et al., 1993); thus, resistance to 4% PEG8000 was another prerequisite for the cell line to ensure productive HBV infection. Therefore, this aspect of Huh7D cells was tested and further confirmed by comparison with HepG2 cells, the platform for an existing HBV infection cell model (HepG2$^{hNTCP}$) (Yan et al., 2012; Ni et al., 2014). As shown in Figure 1B, there were no significant differences between the presence and absence of PEG8000 for all time points except the first day, indicating that Huh7D cells were tolerant to 4% PEG8000. By comparison, the ratio of cell viabilities of Huh7D cells in the presence of PEG8000 was significantly higher than that of HepG2 cells at the indicated time points, implying that Huh7D cells had better resistance to PEG8000 than HepG2 cells (Figure 1C). Thus, Huh7D cells, which were resistant to 2.5% DMSO / 4% PEG8000, were used to establish an hNTCP-expressing cell model.

Figure 1. Resistance to incubation with 2.5% DMSO and 4% PEG8000. (A) Four strains of Huh7 (#1, #2, #3, #4) cells were incubated with 2.5% DMSO for the indicated time points, and viabilities were then measured by CCK8 assay. (B) Huh7D cells were cultured in the presence or absence of 4% PEG8000 for the indicated times, and cell viabilities were measured with ELISA by using CCK8. (C) Huh7D and HepG2 cells were maintained in the presence or absence of 4% PEG8000 for the indicated times. Cell viabilities were measured with ELISA by using CCK8. Data are presented as the ratio of viability in the presence of PEG8000 (+PEG) to viability in the absence of PEG8000 (-PEG). Differences were judged to be statistically significant when $P < 0.05$ (*), $P < 0.01$ (**), and $P < 0.001$ (***).

## Establishment of Huh7D$^{hNTCP}$ cells stably expressing hNTCP

In view of the high infection and integration rate of lentiviruses (Wiznerowicz and Trono, 2003), hNTCP-expressing recombinant lentivirus were produced to efficiently achieve stable hNTCP expression. The hNTCP-expressing pWPI-hNTCP-Puro vector (Figure 2A) was constructed and co-transfected with packaging plasmids (pCMV-dR8.91 and pMD.2G) into the 293T cell line to produce the hNTCP-expressing recombinant lentivirus. The relative mRNA level of hNTCP in Huh7D$^{hNTCP}$ cells was comparable to that in normal human liver tissue but significantly higher than that in Huh7D, HepG2 and HepG2$^{hNTCP}$ cells, a HepG2 cell line that stably expresses hNTCP (Figure 2B). Smearing bands from 55 kDa to 72 kDa corresponding to hNTCP with varied degrees of N-glycosylation could be detected in Huh7D$^{hNTCP}$ cells, which was further validated by the observation that only one 38 kDa band corresponding to nonglycosylated hNTCP could be detected after PNGase F digestion (Figure 2C). Accordingly, the relative protein level of hNTCP in Huh7D$^{hNTCP}$ cells was also comparable to that in normal human liver tissue, whereas the hNTCP level was significantly lower in HepG2$^{hNTCP}$ cells and undetectable in both Huh7D and HepG2 cells (Figure 2D). The immunofluorescence (IF) staining results showed that hNTCP was mainly located at the cell membrane of Huh7D$^{hNTCP}$ and HepG2$^{hNTCP}$ cells (Figure 2E).

## Huh7D$^{hNTCP}$ cells became well differentiated under DMSO treatment

To determine whether Huh7D$^{hNTCP}$ cells could become well differentiated under DMSO treatment, morphological changes, cell cycle progression, and expression of differentiation-associated genes were evaluated. Huh7D$^{hNTCP}$ cells started to undergo progressive morphological changes under DMSO treatment. On day 3 post-DMSO treatment, Huh7D$^{hNTCP}$ cells were induced to form polygonal cells. On day 15 post-DMSO treatment, the morphology of Huh7D$^{hNTCP}$ cells resembled that of PHHs, as both contained a centrally located nucleus, prominent nucleolus, and granular cytoplasm (Figure 3A). The flow cytometry results showed that 83.4%, 2.4%, and 11.6% of Huh7D$^{hNTCP}$ cells were in G0/G1 phase, S phase, and G1/G2 phase, respectively, after applying DMSO for 6 d, whereas approximately 51.6%, 22.6%, and 19.1% of cells without DMSO treatment were in G0/G1, S, and G1/G2 phase, respectively (Figure 3B). The transcription of differentiation-associated genes was examined, such as albumin, α-fetoprotein (AFP), hepatocyte nuclear factor 4α (HNF4α), human cytochrome P450 3A4 (CYP3A4), and asialoglycoprotein receptor (ASGPR). The representative genes positively associated with differentiation (albumin, HNF4a, CYP3A4, and ASGPR) were up-regulated, whereas the gene negatively associated with differentiation (AFP) was down-regulated (Figure 3C). Thus, Huh7D$^{hNTCP}$ cell could be induced into non-dividing, well-differentiated polarized cells under 2.5% DMSO treatment.

## Infection of Huh7D$^{hNTCP}$ cells by HBV

Considering that Huh7D$^{hNTCP}$ cells stably expressed hNTCP and well differentiated into the polarized morphology resembling PHHs under DMSO treatment, we determined whether Huh7D$^{hNTCP}$ cells were permissive for HBV infection. The cells were inoculated with the supernatant of HepAD38 cells stably producing infectious HBV at a MOI of 2000 genome equivalent (GE) per cell. HBeAg and HBsAg secretion gradually increased over time (Figure 4A, 4B). IF staining of intracellular HBcAg and HBsAg was positive in HBV-infected Huh7D$^{hNTCP}$ cells but not Huh7D cells, and approximately 50% of Huh7D$^{hNTCP}$ cells were infected at 11 dpi (Figure 4C).

Subsequently, HepG2$^{hNTCP}$ cells (Yan et al., 2012) were used as a positive control to assess the susceptibility of Huh7D$^{hNTCP}$ cells to HBV infection. Both cell lines were infected with HBV at the indicated MOI, and HBeAg secretion by Huh7D$^{hNTCP}$ cells was higher than that of the positive control (HepG2$^{hNTCP}$ cells). Notably, HBeAg was detectable in the supernatant of infected Huh7D$^{hNTCP}$ cells but was undetectable in the supernatant of infected HepG2$^{hNTCP}$ cells when 40 MOI of HBV was used for inoculation (Figure 4D). At 1000 MOI, HBeAg and HBsAg levels in the supernatant of infected Huh7D$^{hNTCP}$ cells were both significantly higher than those in HepG2$^{hNTCP}$ cells, whereas the entry inhibitor Myrcludex B blocked the infection, proving reliable entry specificity (Figure 4D–4F). At 7 dpi, significant HBV replicating intermediates could be detected in Huh7D$^{hNTCP}$ cells, whereas very weak signals corresponding to viral DNA was observed in HepG2$^{hNTCP}$ cells (Figure 4G). Significantly more HBV transcripts, especially the 3.5 kb RNA, were observed in Huh7D$^{hNTCP}$ cells than in HepG2$^{hNTCP}$ cells (Figure 4H). IF staining also revealed that approximately 25% of Huh7D$^{hNTCP}$ cells were HBcAg positive, whereas only 5% of HepG2$^{hNTCP}$ cells were HBcAg positive (Figure 4I). These data indicated that Huh7D$^{hNTCP}$ cells were a productive cell model for HBV infection.

Considering that Huh7D$^{hNTCP}$ cells expressed higher levels of hNTCP than HepG2$^{hNTCP}$ cells (Figure 2B, 2D), we next determined whether HBV binding and/or uptake differed between these two cell lines. When incubated at 4 °C, more HBV was absorbed by Huh7D$^{hNTCP}$ cells than by HepG2$^{hNTCP}$ cells (Figure 4J). When incubated at 37 °C followed by trypsin treatment, a positive signal could be detected in Huh7D$^{hNTCP}$ cells, whereas there was no detectable signal in HepG2$^{hNTCP}$ cells. Thus, Huh7D$^{hNTCP}$

cells exhibited higher susceptibility to HBV infection than HepG2$^{hNTCP}$ cells probably due to higher HBV entry efficiency. Taken together, these data indicated that Huh7D$^{hNTCP}$ cells were susceptible to HBV infection and could support the entire productive HBV life cycle.

In addition, Huh7D$^{hNTCP}$ cells, but not HepG2$^{hNTCP}$ cells, supported HCV entry (J399EM, genotype 2a) (Supplementary Figure S2) in accordance with the fact that Huh7 cells are fully permissive while HepG2 cells are resistant to HCV infection (Sainz and Chisari, 2006; Mee et al., 2009). Thus, these results indicated that Huh7D$^{hNTCP}$ cells are appropriate for use to investigate the co-infection of HBV and HCV.

## DISCUSSION

It has been well documented that cell polarization and differentiation are crucial for HBV entry and HBV transcription (Gripon et al., 2002; Schulze et al., 2012). For example, PHHs, the native host for HBV infection, rapidly dedifferentiate and lose susceptibility to HBV infection after being isolated and seeded (Zhou et al., 2014a). DMSO treatment can maintain PHH differentiation and susceptibility to HBV infection. Some poorly differentiated human hepatoma derived cell lines such as HepaRG cells can be induced and differentiated into hepatocyte-like cells by DMSO (Gripon et al., 2002). Moreover, DMSO treatment may directly favor HBV transcription and replication (Gripon et al., 1988; Gripon et al., 2002; Schulze-Bergkamen et al., 2003). The expression of hNTCP can also be induced and maintained at a relatively high level by DMSO treatment (Ni

Figure 2. Establishment of Huh7D$^{hNTCP}$ cells stably expressing hNTCP. (A) A cassette of a recombinant lentiviral vector encoding hNTCP was constructed, and the recombinant lentivirus was produced. hNTCP-expressing Huh7D cells were established by lentivirus transduction followed by puromycin screening. (B) Different cell lines were subjected to RT-qPCR to detect relative hNTCP mRNA levels. (C) Cell lysates of Huh7D$^{hNTCP}$ cells were incubated at 37 °C for 1 h in the presence or absence of PNGase F, followed by western blot. (D) Different cell lines were subjected to western blot to detect the relative hNTCP protein levels. The relative protein levels (both glycosylated and non-glycosylated) are depicted as the relative integrated density (Rel. Den.). (E) Different cell lines were seeded at the same density and fixed for immunofluorescence analysis with the indicated antibodies using confocal microscopy, with green for hNTCP and blue for the nucleus. LTR: long tendon repeat; EF-1α: human elongation factor-1α promoter; IRES: internal ribosome entry site; Puro: puromycin-resistance gene.

Figure 3. Characterization of DMSO-treated Huh7D$^{hNTCP}$ cells. (A) Huh7D$^{hNTCP}$ cells were seeded and maintained in the presence of 2.5% DMSO, and PHHs were maintained in 2% DMSO; the morphologies of both cell types were assessed at the indicated time points using a phase-contrast microscope. (B) Huh7D$^{hNTCP}$ cells were induced with 2.5% DMSO for 6 d, and cell cycle progression was analyzed by propidium iodide (PI) staining using flow cytometry. (C) Relative mRNA levels of albumin, α-fetoprotein (AFP), hepatocyte nuclear factor 4a (HNF4a), human cytochrome P450 3A4 (CYP3A4), and asialoglycoprotein receptor (ASGPR) in DMSO-treated Huh7D$^{hNTCP}$ cells were detected by RT-qPCR using the $\triangle\triangle C_T$ method (calibrator Huh7D).

et al., 2014). Therefore, the addition of DMSO at different levels helps to improve HBV infection efficiency. However, high concentrations of DMSO are cytotoxic to hepatocytes (Sumida et al., 2011) and thus interfere with viral infection. In the present study, a Huh7 cell line named Huh7D was found to be tolerant to 2.5% DMSO and to fully differentiate into PHH-like cells under DMSO treatment, which may be the foundation of the successful establishment of productive HBV infection in the Huh7D$^{hNTCP}$ cell line.

A
HBeAg secretion OD450−OD630 (5-fold dillution)
Huh7DhNTCP
Huh7D
Cutoff
Days post infection
B
HBsAg secretion OD450−OD630
Huh7DhNTCP
Huh7D
Cutoff
Days post infection
D
HBeAg secretion (OD450/COV)
Huh7DhNTCP
HepG2hNTCP
Cutoff
Multiplicity of infection
C
DAPI
HBcAg
HBsAg
Merge
Huh7DhNTCP
Huh7D
200 μm
E
HepG2hNTCP
HeG2hNTCP+MyrB
Huh7hNTCP
Huh7hNTCP+MyrB
HBeAg secretion OD450−OD630
Days post infection
F
HepG2hNTCP
HeG2hNTCP+MyrB
Huh7hNTCP
Huh7hNTCP+MyrB
HBsAg secretion OD450−OD630
Days post infection
G
HepG2hNTCP
Huh7DhNTCP
Southern blot
RC
DL
IB
GAPDH
H
HepG2hNTCP
Huh7DhNTCP
dpi
11+MyrB
Northern blot
3.5kb
2.4kb
2.1kb
28S
18S
I
HBcAg
DAPI
Merge
HepG2hNTCP
Huh7DhNTCP
200 μm
J
4 °C
37 °C
Trypsin
MyrB
Int.Den. /10⁴
4.2 7.0 0.0 0.0 2.5 1.2 7.5 14.2
HepG2hNTCP
Huh7DhNTCP

Figure 4. Susceptibility of Huh7D$^{hNTCP}$ cells to HBV infection. Huh7D$^{hNTCP}$ and Huh7D cells were infected with the culture supernatant of HepAD38 cells (MOI = $2×10^3$) for 24 h, and media were collected at 3, 5, 7, 9, and 11 dpi for detection of HBeAg (A) and HBsAg (B) by ELISA. Values over the cutoff line were regarded as positive. (C) Cells at 11 dpi were fixed to detect intracellular HBcAg (green) and HBsAg (red) by IF. Huh7D$^{hNTCP}$ and HepG2$^{hNTCP}$ cells were maintained in 2.5% DMSO for 24 h and infected by HBV at the indicated MOI for 24 h in the presence of 4% PEG8000. (D) HBeAg in the culture medium (11 dpi) was measured by ELISA. To allow the detection of other hallmarks of HBV infection, further experiments were performed at a MOI of 1000 with Myrcludex B (MyrB, 500 nmol/L), which served as a control for infection specificity. Supernatants were collected at the indicated time points for the measurement of HBeAg (E) and HBsAg (F) by ELISA. (G) Intracellular HBV replicating intermediates were extracted from $2 × 10^6$ infected cells at 7 dpi and detected by Southern blot, and the protein level of GAPDH was served as an internal control. (H) Approximately 30 μg total RNA was subjected to northern blot, and 28S/18S served as an internal control. (I) Cells at 11 dpi were fixed to detect intracellular HBcAg (green) and nuclear (blue) by IF. (J) Assay of binding and/or uptake was performed using $2 × 10^6$ cells, which were incubated with the same MOI at 4 °C (HBV binding only), 37 °C (binding and uptake), or 37 °C but digested with trypsin for 10 min (uptake only). MyrB (500 nmol/L) was applied to block HBV entry. The viral DNA was extracted and detected by Sothern blot. Integrated density (Int.Den.) was calculated using Image J software. COV, cutoff value; RC, relax circular HBV DNA; DL, double-stranded HBV DNA.

Next, we stably expressed hNTCP in Huh7D cells and demonstrated that Huh7D$^{hNTCP}$ cells were permissive to HBV infection under DMSO treatment. Compared to another hNTCP stably expressing cell line (HepG2$^{hNTCP}$), Huh7D$^{hNTCP}$ cells exhibited better susceptibility to HBV infection (Figure 4D–4I). This difference in HBV susceptibility was not likely caused by DMSO cytotoxicity because both cell lines were tolerant to 2.5% DMSO (data not shown). Given that Huh7D$^{hNTCP}$ cells expressed more hNTCP than HepG2$^{hNTCP}$ cells (Figure 2B, 2D), we then compared the difference in viral adsorption and entry between these two cell lines. The results showed that Huh7D$^{hNTCP}$ cells exhibited more HBV binding and/or uptake (Figure 4I), which may partially explain the different infectivity levels between these two cell lines. In addition, although both Huh7 and HepG2 cells are hepatoma cell lines, they have distinct expression profiles of genes in various categories (Kawai et al., 2001). Some unknown co-receptors or co-factors besides hNCTP, as well as DMSO-induced factors, necessary for productive HBV infection may be present at different levels in these two cell lines, which may be responsible for the differences in viral entry. Moreover, differences in viral transcription and replication caused by different liver-enriched genes cannot be completely ruled out.

In 2014, Ni *et al*. established the Huh7$^{hNTCP}$ cell line that constitutively expresses hNTCP, using a lentiviral vector. Comparative infection experiments were performed between Huh7$^{hNTCP}$ and HepG2$^{hNTCP}$ cells. When both cell lines were inoculated with $1×10^4$ MOI of HBV stock, Huh7$^{hNTCP}$ cells exhibited weak susceptibility to HBV infection compared to HepG2$^{hNTCP}$ cells under 2.5% DMSO treatment, with a small proportion of cells infected (Ni et al., 2014). However, in the present study, approximately 50% of Huh7D$^{hNTCP}$ cells were infected at a MOI of 2000, and they were more susceptible than HepG2$^{hNTCP}$ cells. Thus, it is still uncertain whether Huh7 cells are truly resistant to HBV compared to HepG2 cells. More studies are needed to determine why Huh7D$^{hNTCP}$ cells in the present study were different from other established Huh7$^{hNTCP}$ cell lines. In addition, a limitation of this study was that only one strain of HBV (HepAD38, genotype D) was tested in this model because HepAD38 has been widely used for the vigorous production of HBV virions. However, there are eight genotypes of HBV (Schaefer, 2007) and different mutants, which may cause varying degrees of clinical viral hepatitis. It is possible that other HBV strains, e.g., other genotypes and/or HBeAg-negative isolates, could behave differently in terms of viral protein expression and infectivity, which need to be further studied. Nevertheless, the establishment of the Huh7D$^{hNTCP}$ cell line as another option will give virologists an alternative cell model to study HBV entry and develop novel antiviral agents.

## ACKNOWLEDGMENTS

We gratefully acknowledge Prof. Li Wenhui from National Institute of Biological Sciences (Beijing) for his kindly providing HepG2$^{hNTCP}$, anti-HBc antibody and protocols. This work was supported by the National Natural Science Foundation of China (Grant number: 81601760, 31621061 and 81461130019), General Financial Grant from the China Postdoctoral Science Foundation (Grant number: 2016M602587) and the Shenzhen Foundation of Science and Technology (Grant number: JCYJ20160425 104534335). Chunchen Wu is supported by the Youth Innovation Promotion Association CAS (No.201603).

## COMPLIANCE WITH ETHICS GUIDELINES

The authors declare that they have no conflict of interest. Additional informed consent was obtained from all participating family members, who donated human materials. Ethical approval for the study was granted by the institutional bioethics committee of Wuhan Institute of Virology, Chinese Academy of Sciences (approval number WIVH24201101).

## AUTHOR CONTRIBUTIONS

CCW and MZ designed the study. MZ performed most of the experiments; KT Z, YXY, YF Y, XH and YZ participated in the experiments; RJP, YW and JZC analyzed the data. MZ, XWC and CCW wrote and finalized the manuscript. All authors read and approved the manuscript.

Supplementary figures are available on the websites of *Virologica Sinica*: www.virosin.org; link.springer.com/journal/12250.

## OPEN ACCESS

This article is distributed under the terms of the Creative Commons Attribution 4.0 International License (https://creativecommons.org/licenses/by/4.0/), which permits unrestricted use, distribution, and reproduction in any medium, provided you give appropriate credit to the original author(s) and the source, provide a link to the Creative Commons license, and indicate if changes were made.

## Electronic supplementary material

Productive HBV infection of well-differentiated, hNTCP-expressing human hepatoma-derived (Huh7) cells
