# Supplementary material for: Productive HBV infection of well-differentiated, hNTCP-expressing human hepatoma-derived (Huh7) cells
Source: Virol Sin. 2017 Sep 29;32(6):465–75. doi: 10.1007/s12250-017-3983-x (PMC6702241; doi:10.1007/s12250-017-3983-x)
Supplement: Supplementary file 1 — Productive HBV infection of well-differentiated, hNTCP-expressing human hepatoma-derived (Huh7) cells [file 12250_2017_3983_MOESM1_ESM.pdf]

Electronic Supplementary Material

Productive HBV infection of well-differentiated, hNTCP-expressing human hepatoma-derived (Huh7) cells

Ming Zhou[1,2,3], Kaitao Zhao[1], Yongxuan Yao[1], Yifei Yuan[1], Rongjuan Pei[1], Yun Wang[1], Jizheng Chen[1], Xue Hu[1], Yuan Zhou[1], Xinwen Chen[1], Chunchen Wu[1]✉

1. State Key Laboratory of Virology, Wuhan Institute of Virology, Chinese Academy of Sciences, Wuhan 430071, China
2. Shenzhen Xenotransplantation Research and Development Center, State and Local Joint Cancer Genome Clinical Application of Key Technology Laboratory, Shenzhen Second People's Hospital, First Affiliated Hospital of Shenzhen University, Shenzhen 518035, China
3. Institute of Immunology, Zhongshan School of Medicine, Guangdong Provincial Key Laboratory of Organ Donation and Transplant Immunology, Sun Yat-sen University, Guangzhou 510080, China

Supporting information to DOI: 10.1007/s12250-017-3983-x

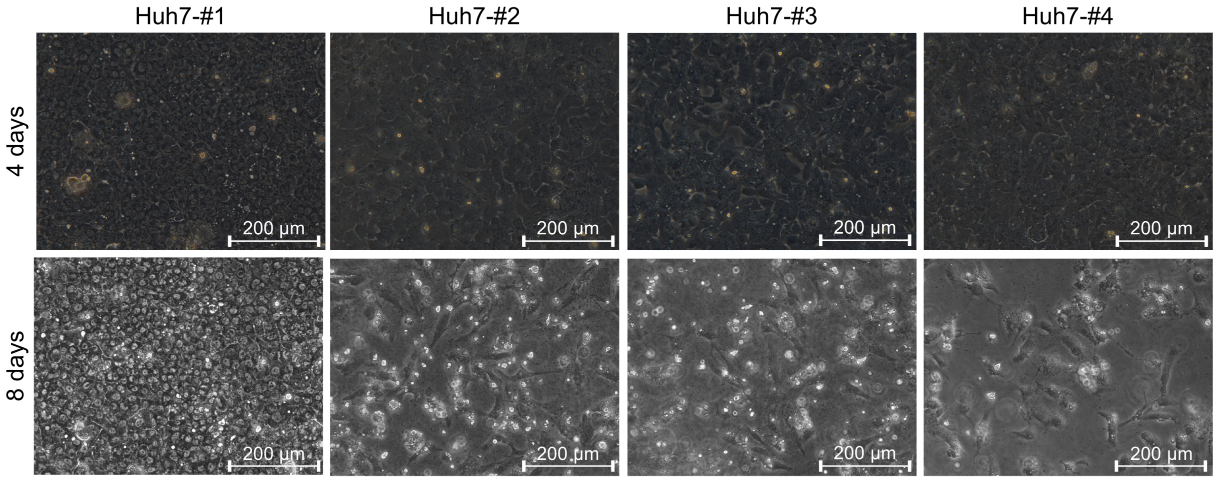

Figure S1. Morphological characteristics of cell lines following DMSO treatment. A panel of Huh7 cell lines (#1–#4) was seeded at 1.0×10^5 cells/well in a 12-well culture plate and maintained in 2.5% DMSO. The #1, #2, and #4 Huh7 cells were obtained from separate labs, and the #3 Huh7 cells were obtained from the China Center for Type Culture Collection (CCTCC). Morphological changes were recorded at 4 and 8 d using a phase-contrast microscope.

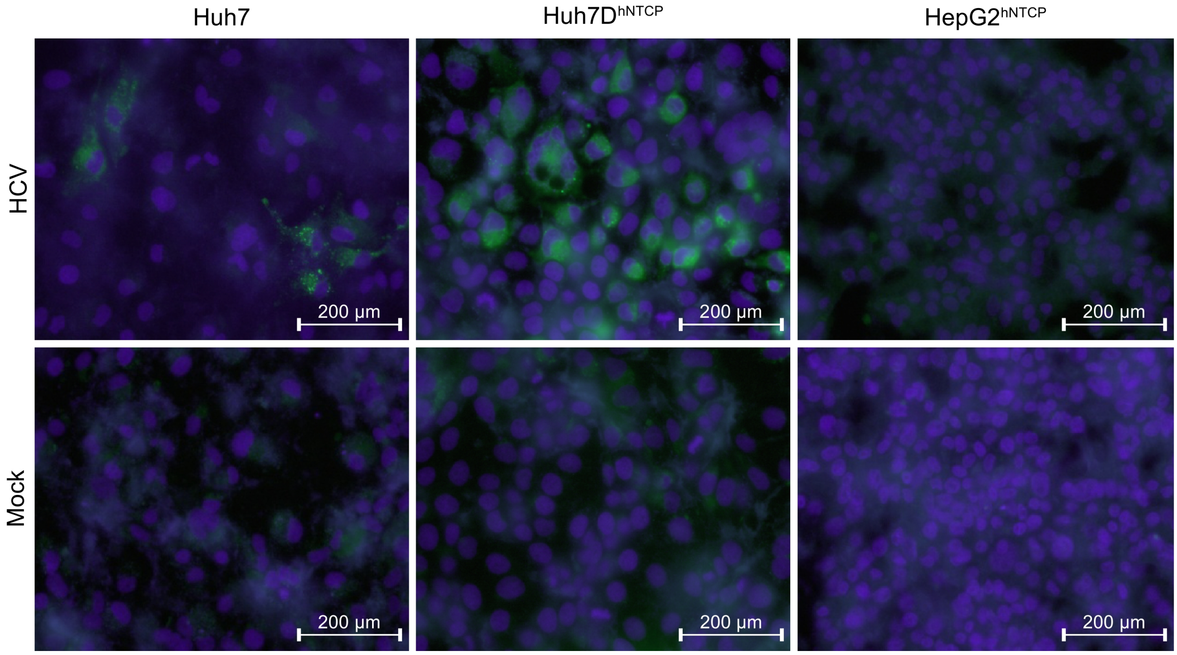

Figure S2. HCV infection of Huh7D^{hNTCP} cells. Huh7, Huh7D^{hNTCP}, and HepG2^{hNTCP} cells were seeded at 50% confluence and infected by J399EM (genotype 2a, MOI = 1) and maintained in the presence of 2.5% DMSO. Cells were fixed at 4 dpi and stained with DAPI. Fluorescent signals were recorded under a fluorescence microscope, with green indicating chimeric HCV-GFP and blue indicating the nucleus.
